# Double Trouble: A Case of an Evolving Leg Ulcer in the Setting of Heart Failure

**DOI:** 10.7759/cureus.80028

**Published:** 2025-03-04

**Authors:** Osman Zuberi, Micah Pippin, Diahann Marshall

**Affiliations:** 1 Family Medicine, Louisiana State University Health Sciences Center, Alexandria, USA

**Keywords:** chronic venous insufficiency (cvi), chronic venous leg ulcers, congestive heart failure (chf), venous stasis ulcer, wound care management

## Abstract

Congestive heart failure can result in low perfusion to the body's periphery and fluid pooling in the lower extremities. Investigators have suggested that this combination of factors can cause venous leg ulcers to deteriorate, although reports of this association are limited in the literature. We present a case of a lower extremity venous ulcer, which worsened concomitantly with an acute exacerbation of chronic congestive heart failure, eventually requiring surgical debridement. This case report serves to investigate the assertion that comorbid heart failure, especially during acute exacerbation, may worsen wound healing in lower extremity venous ulcers.

## Introduction

Venous leg ulcers (VLUs) are manifestations of advanced chronic venous insufficiency (CVI). In advanced stages, chronic venous insufficiency leads to venous valve incompetence, which can cause retrograde blood flow, obstruction, or both. It is well-established that venous insufficiency is associated with cardiac abnormalities. Although chronic heart failure (CHF) is a prevalent cardiac disease and has been suggested to be a risk factor for developing venous ulcers, recent studies have found no bidirectional causal relationships between the two conditions and have recommended further investigation [1]. This report presents a previously consistently improving venous ulcer showing stagnation and deterioration during an acute CHF exacerbation. The ulcer became infected, and the healing process was hindered, thus prompting operative debridement. This case suggests that acute CHF exacerbations may play a role in venous ulcer decompensation and lends credence to the possibility of a causal relationship between the two pathologies [2].

This case describes a patient who was admitted to the inpatient unit for an acute CHF exacerbation and management of a chronic venous lower extremity ulcer. During hospitalization, there was an acute but significant change in the ulcer's wound bed composition, which occurred synchronously with the patient's symptomatic and hemodynamic decrease in cardiac function. The reported case brings into question a potential association between CHF and venous ulcers and may support the importance of a multidisciplinary approach when treating venous ulcers, one that not only focuses on direct wound care through topical dressings and debridements but also on systemic maintenance of cardiac function.

## Case presentation

**Figure 1:**
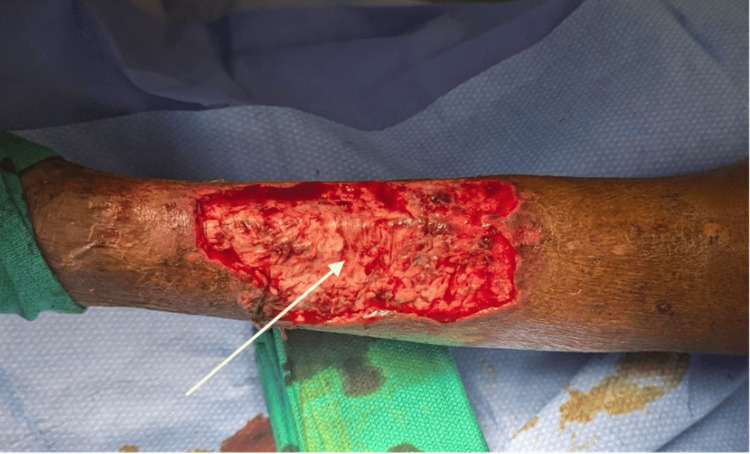
Initial presentation of the left lower extremity ulcer

A 78-year-old African American female presented to the emergency department complaining of chest congestion and shortness of breath. Her past medical history was significant for obesity, diabetes mellitus type 2, coronary artery disease, chronic atrial fibrillation, congestive heart failure with a mid-range ejection fraction of 45%, and chronic kidney disease (CKD) stage two. Her past surgical history included a coronary artery bypass graft (CABG) several years prior. The patient reported a remote smoking history of five pack years in her 30s but did not endorse any alcohol consumption or illicit drug use. Before her presentation, the patient's baseline was ambulatory with mild dyspnea upon ordinary activity (New York Heart Association NYHA Class 2) and with a largely sedentary lifestyle. Her lower extremity venous ulcer was stable and managed with graded compression stockings and leg elevation at bedtime. Her chronic medications included the angiotensin receptor blocker (ARB) losartan at 50 mg by mouth daily, the biguanide metformin at 500 mg by mouth twice daily, and the loop diuretic furosemide at 20 mg orally daily. She did report that she had not taken her furosemide for the last two weeks. The patient's dyspnea was worsened with exertion and by lying flat. There was no associated cough or fever with her shortness of breath. She did not complain of chest pain or palpitations but did report worsening of her chronic lower extremity swelling. On physical examination, she appeared to be hypervolemic, given her jugular vein distension (JVD), worsening bilateral lower extremity edema, orthopnea, and crackles on bilateral lung auscultation. Skin assessment was significant for a 14.5 x 9 x 0.1-centimeter venous ulcer lower on the anterior, distal aspect of her left lower extremity (Figure 1).

The ulcer presented with minimal serosanguinous exudate with granulation tissue and minimal slough. No induration, purulence, malodor, or surrounding erythema was noted during the initial inspection. The patient stated that she had first noticed the ulcer approximately five months before this admission, and it was improving with wound care and compression stockings. She was admitted to the hospital for diuresis of her heart failure-associated edema and wound care and surgical management of her venous ulcer.

A Doppler ultrasound confirmed venous insufficiency but did not reveal any evidence of deep vein thrombosis (DVT) in the affected limb. Surgical and wound care orders included rinsing the ulcer with sterile normal saline, applying a Mepilex® transfer Ag adhesive foam dressing to the ulcer base with prophylactic skin prep to the peri-wound, and securing the dressing with kerlix roll gauze. The patient was encouraged to elevate her legs at nighttime. Given the venous ulcer’s apparent stable condition, the focus of her hospitalization became the optimization of her cardiac and renal function. The plan was to establish a follow-up for the venous ulcer and perform ankle-brachial index (ABI) testing as an outpatient.

**Figure 2:**
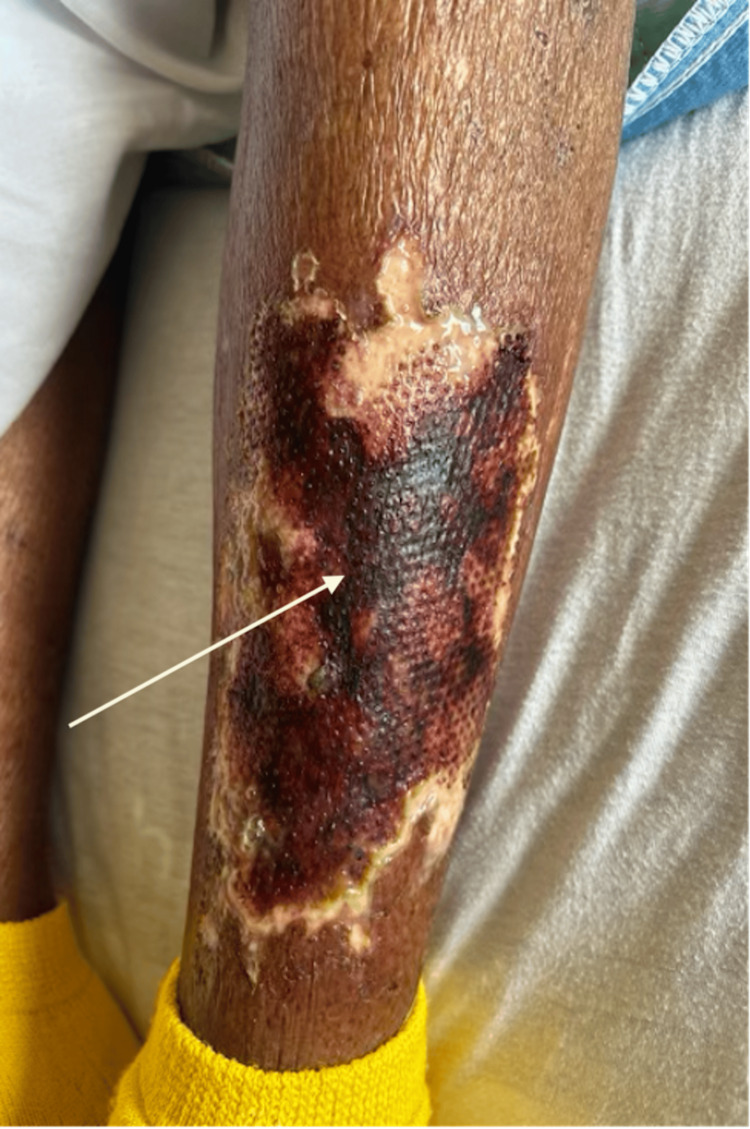
Progressing left lower extremity venous ulcer with extensive eschar tissue

Subsequently, during admission, the patient's cardiac decompensation progressed with acute-on-chronic renal failure from cardiorenal syndrome, and the venous ulcer grew to 15.5 x 8.8 x 0.2 cm. The wound developed moderate serosanguinous exudate and extensive eschar tissue, and there was an acute change in wound bed composition when compared to that on admission (Figure 2).

Furthermore, the patient became much more somnolent, and staff reported altered mental status and new-onset pain out of proportion at the ulcer site. A TheraHoney® dressing was applied to the ulcer and secured by kerlix roll gauze. The surgery team was re-consulted and recommended immediate surgical debridement in the operating room following initiation of hemodialysis for worsening renal function and persistent lower extremity edema. The eschar tissue from the ulcer was debrided, and an amniotic membrane was applied to the post-op surgical wounds to aid the wound-healing process. Operative wound cultures revealed a Pseudomonas aeruginosa infection that was sensitive to fluoroquinolones. She was placed on a 14-day course of oral ciprofloxacin 500 mg twice a day with daily dressing changes. The patient's overall clinical status improved with dialysis, resolving her confusion and pain and decreasing her extremity fluid burden. With the improvement of her volume status, the venous ulcer stabilized and started healing. She was discharged to a skilled nursing facility for rehabilitation, ongoing dialysis, and local wound care.

## Discussion

Venous insufficiency results from extensive inflammation within the venous circulation and venous hypertension, eventually leading to dermatological and vascular complications associated with venous ulcers [3-4]. Venous ulcers account for up to 80% of all ulcers affecting the lower extremities and at least 60% of chronic ulcers [4]. The incidence of venous ulcers is increased among females, and African American patients often present with more advanced venous disease at younger ages [4-5]. Established risk factors for venous insufficiency and venous ulcers include old age, obesity, inactivity, smoking, diabetes, previous leg injury, history of deep vein thrombosis, and atrial fibrillation [5-8]. While general cardiovascular disease has been associated with venous insufficiency and ulcer formation, a direct correlation between congestive heart failure and the development and worsening of venous ulcers has not been clearly established.

Venous insufficiency generally presents with dependent lower extremity edema, leg heaviness and discomfort, and itching. Symptoms often worsen with activity throughout the day and improve with rest [8-9]. Later stages of venous insufficiency can produce findings such as varicose veins (enlarged venous blood vessels), corona phlebectatica (visible veins and telangiectasias of the ankle and foot), and lipodermatosclerosis (painful skin thickening and induration), resulting in the classic champagne bottle leg appearance [8]. Skin darkening and ulcers are manifestations of chronic venous insufficiency progression [8-9]. Venous ulcers are predominantly found on the medial side of the lower extremity near the malleolus. They are characterized by irregularly shaped, shallow wounds with well-defined borders and surrounding skin hyperpigmentation [9].

Initial evaluation of suspected venous insufficiency and venous ulcers should include lower extremity venous Doppler ultrasonography to investigate for reflux and obstruction [8-9]. Physical examination for arterial pulses, an ankle-brachial index, and possible arterial ultrasound should be performed to assess for peripheral artery disease (PAD), an important differential diagnosis for venous insufficiency and a frequent comorbid condition [9]. It is crucial to exclude PAD as compressive treatment of venous insufficiency and venous ulcers is contraindicated in peripheral artery disease due to the risk of ischemia [8-9]. When the diagnosis of venous insufficiency or a venous ulcer is in question, a skin biopsy can be performed, which may show dermal fibrosis, dilated capillaries, inflammation, and increased hemosiderin pigment deposition [9].

Compression therapy is the cornerstone of venous insufficiency and ulcer management, which may include wraps, bandages, or graduated stockings [3,4,9]. Increased physical activity, specifically prescribed exercise and resistance training, has demonstrated effectiveness for venous wound healing [3,8-9]. Wound care and dressings are recommended; however, no individual dressing has proved superior to the others [9]. Pentoxifylline, alone or in combination with compression therapy, can improve rates of venous ulcer wound healing [9]. Phlebotonics, such as horse chestnut seed extract, may improve edema and symptoms of venous insufficiency but do not affect venous ulcer prognosis [9]. Endovenous ablation to correct superficial venous reflux is an evidence-based intervention to improve venous ulcer healing rates [8-9]. One study found that tissue-based products, such as the amniotic membrane utilized in this case, can accelerate venous ulcer healing [8-9]. Skin grafting may be required for large or non-healing venous ulcers. Systemic antibiotics and debridement may be warranted for acutely infected venous ulcers [3,8-9].

Establishing a direct cause-and-effect relationship between heart failure and venous insufficiency and, thereby, a correlation with the worsening of venous ulcers is made difficult by several confounding variables. Heart failure and venous insufficiency have common risk factors, including age, obesity, inactivity, smoking, and hypertension, which could suggest a shared etiology and not a direct causal link [3,10]. The frequently insidious onset of both conditions also makes confirmation of an immediate association burdensome [3]. Logically, the diminished cardiac function in heart failure could result in or worsen venous insufficiency and ulcers by causing accumulation of peripheral fluid and inhibiting venous return; however, it is challenging to confirm direct causality. Suggested mechanisms for heart failure's possible deleterious effects on lower extremity venous health and ulcers may include the decreased physical activity exhibited by patients following their heart failure diagnosis [11]. Orthopnea (dyspnea when supine) from heart failure results in a patient's preference for a seated position, which can lead to dependent edema and worsening fluid accumulation in the lower extremities [11]. Impaired perfusion and hypoxemia associated with decreased cardiac output in heart failure may hinder wound healing in chronic venous ulcers [10]. Heart failure patient's predisposition to thrombotic events may manifest as an increased incidence of venous thromboembolism and, therefore, new or progressive venous insufficiency [10]. Worsening depression commonly present in heart failure patients may contribute to reduced physical activity, exacerbating venous insufficiency and causing delayed wound healing [12]. Compression therapy, a mainstay of venous insufficiency management, may be avoided in New York Heart Association (NYHA) class 3 or 4 heart failure patients due to the potential for adverse effects on cardiac function [13]. Therefore, worsening heart failure may lead to the exclusion of therapeutic interventions for venous insufficiency and ulcers [13].

The association between heart failure and venous insufficiency and the resulting venous ulcers is still unclear in the literature, although recent studies have made some connections. Garavello et al. have identified heart disease as a significant risk factor for venous ulcer incidence, further explaining that treating this comorbidity can heal the ulcer and prevent recurrences [14]. Melikian et al. explained the association between heart failure and delayed venous ulcer healing, although this association was only significant on univariate analysis [15]. Edwards et al. described how the co-occurrence of multiple chronic diseases, including heart failure, can contribute to depression and poor quality of life, which can discourage patients from mobilizing and exercising their calf muscles to improve venous blood flow [12]. These findings collectively suggest a possible correlation between heart failure and venous ulcers and that management may benefit from a holistic approach that focuses not only on venous ulcers but also on their comorbid medical conditions, including heart failure. Further studies are warranted to elucidate the association between heart failure and venous insufficiency and to identify heart failure’s role in venous ulcer progression and delayed resolution.

## Conclusions

This case of a worsening lower extremity venous ulcer in the setting of a patient with heart failure and an acute exacerbation motivates questions of a possible connection between the two conditions. While current literature has not yet established a causal relationship, the idea of heart failure negatively impacting the progression of venous insufficiency and venous ulcers is reasonable based on our understanding of the physiology of both. It remains challenging to confirm this association, but recent studies have advanced our understanding of a possible correlation. Further investigation is required to identify a link between heart failure and venous ulcers definitively. This endeavor could potentially spur future exploration into new holistic management strategies in which optimizing a patient's heart failure may improve outcomes associated with venous insufficiency and venous ulcers.
